# Disruption of White Matter Integrity in Adult Survivors of Childhood Brain Tumors: Correlates with Long-Term Intellectual Outcomes

**DOI:** 10.1371/journal.pone.0131744

**Published:** 2015-07-06

**Authors:** Tricia Z. King, Liya Wang, Hui Mao

**Affiliations:** 1 Department of Psychology & Neuroscience Institute, Georgia State University, Atlanta, Georgia, United States of America; 2 Department of Radiology and Imaging Sciences, Emory University School of Medicine, Atlanta, Georgia, United States of America; Université de Montréal, CANADA

## Abstract

**Background:**

Although chemotherapy and radiation treatment have contributed to increased survivorship, treatment-induced brain injury has been a concern when examining long-term intellectual outcomes of survivors. Specifically, disruption of brain white matter integrity and its relationship to intellectual outcomes in adult survivors of childhood brain tumors needs to be better understood.

**Methods:**

Fifty-four participants underwent diffusion tensor imaging in addition to structural MRI and an intelligence test (IQ). Voxel-wise group comparisons of fractional anisotropy calculated from DTI data were performed using Tract Based Spatial Statistics (TBSS) on 27 survivors (14 treated with radiation with and without chemotherapy and 13 treated without radiation treatment on average over 13 years since diagnosis) and 27 healthy comparison participants. Whole brain white matter fractional anisotropy (FA) differences were explored between each group. The relationships between IQ and FA in the regions where statistically lower FA values were found in survivors were examined, as well as the role of cumulative neurological factors.

**Results:**

The group of survivors treated with radiation with and without chemotherapy had lower IQ relative to the group of survivors without radiation treatment and the healthy comparison group. TBSS identified white matter regions with significantly different mean fractional anisotropy between the three different groups. A lower level of white matter integrity was found in the radiation with or without chemotherapy treated group compared to the group without radiation treatment and also the healthy control group. The group without radiation treatment had a lower mean FA relative to healthy controls. The white matter disruption of the radiation with or without chemotherapy treated survivors was positively correlated with IQ and cumulative neurological factors.

**Conclusions:**

Lower long-term intellectual outcomes of childhood brain tumor survivors are associated with lower white matter integrity. Radiation and adjunct chemotherapy treatment may play a role in greater white matter disruption. The relationships between white matter integrity and IQ, as well as cumulative neurological risk factors exist in young adult survivors of childhood brain tumors.

## Introduction

Advances in diagnosis and treatment have led to improved clinical outcomes and survival of pediatric brain tumor patients [1, 2]. However, the presence of the tumor and ensuing treatment, such as radiation therapy, are known to induce brain injury that causes a concern regarding long-term intellectual outcomes of survivors. Adverse effects of the tumor and the treatment may appear chronically over brain structural and functional development or as late-effects when childhood brain tumor survivors are emerging as adults with increasing functional and cognitive demands. One longitudinal study of intellectual development among pediatric medulloblastoma patients treated with radiation with or without chemotherapy found that these children acquired new information and skills at a lower rate relative to normative data (49–62%) leading to progressive decline in IQ scores over time [3]. As the number of long-term survivors of childhood brain tumor continues to grow, it becomes more important to understand the intellectual abilities of these survivors several years after treatment as well as the possible neural mechanisms related to their cognitive outcomes. One particular interest is the effect of treatment on white matter integrity that is important to both structural and functional connectivity of the brain.

Diffusion tensor imaging (DTI) is a magnetic resonance imaging (MRI) technique sensitive to the directionality and mobility of water diffusion in tissue. It offers great sensitivity and region specificity to subtle and microscopic change in the white matter tissue; even normal appearing white matter (NAWM) in routine T_1_ and T_2_ weighted MRI. Thus, DTI provides an indirect measure of white matter integrity and connectivity [4]. Fractional anisotropy (FA), a commonly derived index from diffusion tensor data, is a measure of the degree of directional restricted motion of water molecules in the white matter tracts. A lower FA value is often interpreted as disrupted white matter integrity when compared to that of healthy and demographically matched controls.

Previous DTI studies of pediatric medulloblastoma patients have reported lower FA in those who received radiation treatment with or without chemotherapy, suggesting that the lower FA reflects damage to existing white matter tracts and disruption of developing white matter tracts due to the neurotoxic effects of radiation and potentially other concurrent tumor-related events (e.g., hydrocephalus, surgery) [5–9]. Multifaceted treatment that includes radiation therapy, chemotherapy, and often endocrine therapy has contributed to increased survival rates in pediatric brain tumor patients. However, most studies that examine outcomes of survivors following radiation therapy with or without chemotherapy highlight the negative sequelae following radiation treatment. In our own research, we have discussed that reporting that the outcomes are due to the neurotoxic effects of radiation may be oversimplified. Taking into consideration co-occurring treatments and sequelae, and quantifying the cumulative nature of these treatment differences as Neurological Predictor Scores (NPS, [10]) is necessary to gain further understanding of this topic particularly when conducting research with complex treatment samples. In the current study we examined a group of survivors who were treated with radiation therapy with or without chemotherapy and compared them to a group of survivors who were treated without radiation therapy and primarily surgery with comparison of each group to a healthy neurotypical demographically-matched control group.

Rarely do studies examine brain tumor survivors treated with radiation versus no radiation treatment [8] and relative to non-clinic referred healthy controls. The reports on cognitive correlations with FA are also limited [11, 12]. Furthermore, existing studies on the relationship between FA and intellectual performance (IQ) are inconsistent in pediatric brain tumor patients [6, 13]. More specifically, it is not clear if intellectual outcomes are related to specific brain white matter pathways, which may be disrupted by the damage related to the tumor and treatment received in childhood when the brain is developing and vulnerable to injury. Even less is known about the white matter integrity of long-term adult survivors of childhood brain tumors. Moreover, earlier studies [14] also cautioned that selection of region of interest (ROI) on FA maps can be biased, although recent development of a method of voxel-based tract-based spatial statistics (TBSS) [15] employed by the current study is able to improve the ROI selections with a data-driven approach.

Here we report the investigation of white matter integrity differences among long-term survivors of childhood brain tumors treated with radiation treatment with and without chemotherapy (RT), those treated without radiation treatment and primarily neurosurgery (NRT), and healthy demographically-matched controls (HC). In addition, the possible relationships between these regions of identified white matter differences and IQ were explored. Childhood brain tumor survivors often have other neurological factors that can be associated with cognitive outcome. These neurological risk factors include radiation treatment, chemotherapy, endocrine dysfunction, hydrocephalus, and extent of neurosurgery. The association of white matter integrity with a cumulative measure of these factors using the Neurological Predictor Scale (NPS) [10, 16] was examined to explore a more nuanced understanding of the impact on white matter integrity. The results from this study provide new evidence of the microstructure disruption and related intellectual long-term outcomes in adult survivors of childhood brain tumors.

## Methods

### Participants

The study was approved by the Georgia State University Institutional Review Board and the GSU / GA Tech Joint Center for Advanced Brain Imaging Institutional Review Board. Twenty-seven long-term adult survivors of childhood brain tumors, including a group of 14 survivors with radiation treatment with and without chemotherapy (RT) and a group of 13 treated with no radiation treatment (NRT), and twenty-seven demographically matched healthy controls (HC) were enrolled in the study after providing signed informed consent. Inclusion of all fifty-four participants was contingent on being a native English speaker, safe for MRI scans, and adequate hearing and vision screens (one survivor is legally blind and did not complete the two performance subtest IQ measures that require vision). The neuroimaging data also were screened to ensure each was free of metal artifact, motion and distortion. Control participants were excluded if they reported any past or present neurological condition or event (e.g., concussion, seizure, migraines) or if they met diagnostic criteria for current Major Depressive Episode, substance abuse or dependence, or psychotic disorder based on SCID-II interview.

Twenty-seven adult survivors of pediatric brain tumor who met the inclusion and exclusion criteria had a mean age of 22.7 years old (SD = 4.5; range = 18–32; 48% female). The average age of diagnosis was 9 years old (SD = 5.14), and the average time between diagnosis and testing was 13.7 years (SD = 5.37). Tumor pathology, location, type of treatment and treatment history after the tumor resection or biopsy were obtained from medical records. Survivors in the NRT group (n = 13) had tumors located in posterior fossa (10), temporal lobe (2), or occipital lobe (1) with tumors diagnosed as astrocytoma (n = 9), oligodendroglial (1), embryonal (1), choroid plexus papilloma (n = 1), and mixed neuronal-glial (n = 1). Survivors in the RT group (n = 14) had tumors located in posterior fossa (11), hypothalamus (1), third ventricle (2) with tumors diagnosed as embryonal (9), astrocytoma (2), craniopharyngioma (2), and pineal (1). Median radiation dose received was 5400 cGY (range: 5040–5940). The HC group (n = 27) consisted of age- and gender-matched neurotypical-control participants recruited from friends of survivors, research participant pools, and community advertisements. Similar demographics across groups (n = 54) are detailed in Table 1.

**Table 1:** Demographic, treatment history, and intellectual performance of each group.

	RT (n = 14)	NRT (n = 13)	Control (n = 27)
**Age at testing (years)**	22.40±4.36	23.62±4.94	22.86±4.31
**Female (%)**	50%	46%	48%
**Right handed (%)**	93%	69%	85%
**Education (years)**	13.36±2.73	14.08±2.36	14.44±1.69
**Age at Diagnosis (years)**	9.00±5.2	9.00±5.29	
**Time since diagnosis (years)**	12.96±4.53	14.44±6.24	
**No/Subtotal/Gross Total Surgery**	1/4/9	0/2/11	
**Hydrocephalus**	11/14	8/13	
**Shunt**	2/7	4/13	
Hormone deficiency*	13/14^a^	2/13^b^	
Chemotherapy*	11/14^a^	1/13^b^	
Neurological Predictor Scale*	7.92 ±1.38^a^	4.15 ±0.80^b^	
Verbal IQ*	94±14.54^a^	106.46±8.72^b^	107.67±8.57^b^
Performance IQ*	97.85±12.69^a^	109.85±8.50^b^	108.81±10.60^b^
Full Scale IQ*	95.46±14.63^a^	109.38±7.47^b^	109.44±7.50^b^
Vocabulary*	43.79a±11.72^a^	54.92±6.22^b^	54.41±5.96^b^
**Similarities**	48.07a±9.74^a^	53.77±6.61^a^ ^,^ ^b^	55.48±6.81^b^
Block Design*	48.62a±8.09^a^	56.92±5.99^b^	54.56±8.12^b^
Matrix Reasoning*	48.54a±11.40^a^	55.54±6.15^b^	56.56±6.04^b^

Note: RT = survivors who received radiation treatment with or without chemotherapy, NRT = survivors who did not receive radiation treatment. Groups were similar across demographic variables.

*: Variables with significant group difference (*p* < .05).

^a,b^: Different superscripts (e.g., ^a^ and ^b^) signify significant mean differences between groups (χ2, *p* < .05), whereas matching superscripts illustrate similar means (e.g., ^b^ and ^b^). RT group had significantly more individuals treated with chemotherapy and individuals identified with hormone deficiency. Across most cognitive tasks and indices, the RT group was significantly lower relative to both NRT and HC groups; in contrast, the NRT group was similar to controls. IQ Mean = 100, SD = 15; Subtest T Score Mean = 50, SD = 10.

### Procedure

Testing took place typically over two visits. At the first visit, trained psychology graduate students (under supervision of a licensed clinician; TK) administered a medical and developmental history interview, a structured clinical interview (SCID-II for DSM-IV TR Axis I [17]), a vision screen, hearing screen with a standard tone audiometer, and Wechsler Abbreviated Scale of Intelligence (WASI) [18] among other measures. The second visit was the neuroimaging exam when anatomical MRI and DTI data were collected. Survivors in the study were interviewed to gather information about medical variables, such as age at diagnosis and treatment history. Medical record review verified these details. NPS [10, 16] was calculated based on these details to quantify treatment and neurological complexity for each participant. The first visit lasted approximately 5 hours and the second one lasted approximately 2 hours including 1 hour of the imaging protocol.

#### Intelligence Quotients

The 4-subtest Wechsler Abbreviated Scale of Intelligence (WASI; Wechsler, 1999) was administered to obtain estimates of intellectual abilities. WASI has been employed in many research studies of child and adult neurological populations including adults survivors of childhood brain tumor [12, 19]. Verbal IQ (VIQ) was based on Vocabulary and Similarities subtests. Performance IQ (PIQ) was based on Block Design and Matrix Reasoning subtests. VIQ and PIQ indices were employed to examine the relationship of verbal and performance IQ with white matter integrity. WASI standard scores were computed using age-based normative data (M = 100, SD = 15) presented in **Table 1**.

#### MRI Data Acquisition

Anatomical MRI and DTI were performed on all participants using a 3T MRI scanner (Siemens TrioTim) and a 12-channel head coil. Axial T_2_ weighted fluid attenuated inversion recovery (FLAIR) imaging was used to evaluate whether white matter abnormalities, such as lesion, edema and infarcts, in the brain using imaging parameters of: TR/TE = 6000/93 ms, flip angle = 130°, inversion time = 2030 ms, slice thickness = 3 mm. A field of view (FOV) of 240 mm and matrix of 512× 512 were used. In addition, three-dimensional high-resolution anatomic sagittal T_1_ weighted magnetization-prepared rapid gradient-echo (MPRAGE) imaging was performed with TR/TE = 2250/3.98 ms, degree of flip angle = 9, inversion time = 850 ms, slice thickness = 1 mm, FOV of 256 mm and matrix of 256× 256. These T_1_ weighted images were used not only for evaluation of abnormalities, but they also provide anatomic information for DTI and data analysis.

For DTI, the same FOV and slice locations used in the T_1_ weighted structural MRI were applied so that the images could be aligned with structural images. Images were recorded in the axial direction with 60 slices and 2 mm thickness without gap. Directional sensitized diffusion weighting single-shot spin echo echo-planar imaging (EPI) sequence with 30 gradient directions was used with imaging parameters: TR/TE = 7700/90 ms, flip angle = 90°, *b*-values of 0 or 1,000 s/mm^2^ using the b = 0 image as a reference.

#### Image Data Processing and Analysis

High resolution anatomic T_1_ weighted images and T_2_ weighted FLAIR images from all participants were reviewed by an experienced neuroradiologist (LW). All controls and adult survivors of childhood brain tumors had NAWM in supratentorial regions with the exception of some minor white matter hyperintensity. Regions with white matter abnormalities identified on T_1_ weighted and FLAIR images that also were located in the TBSS identified white matter ROIs were not included in the subsequent analyses, so that analysis of the correlation of FA measurement with IQ were based only on NAWM. Diffusion tensor images were processed and analyzed using the FMRIB Software Library version 5.0.1 (FSL, Oxford University, UK www.fmrib.ax.ac.uk/fsl; [20]). For whole brain analysis, TBSS (V.5.0.1) was used to find differences in FA between different groups throughout the white matter of the entire brain [15]. We used the randomizing program within FSL to carry out permutation-based testing. A statistical threshold-free cluster enhancement (TFCE) analysis was implemented from randomize [21, 22] with a cluster *P* < 0.05 (with Family Wise Error Correction). Voxels in the white matter skeletal mask obtained from TBSS were isolated and labeled to include the full width of the white matter tract if they were found to be significantly different between groups. Then, specific ROIs were created using the mask of the clustered voxels to perform quantitative analyses of FA values for individual participants. Significant regions were defined by a cluster of voxels (n > 100) [23]. Measurements of FA were obtained in different clusters of each individual using voxel-wise statistical analysis based on the tracts determined in TBSS, then were used in group comparison analysis. It was predicted that whole brain TBSS would identify the specific ROIs of difference. The most probable anatomic localization of each cluster was determined by FSL atlas tool (http://www.fmrib.ox.ac.uk/fsl/data/atlas-descriptions.html/). The white matter tracts were smoothed with a 3 mm Gaussian kernel, while maintaining the maximum statistic values and limiting the extent of the spread to voxels in the white matter (defined as FA > 0.2).

#### Statistical Analysis

Analyses were conducted with IBM SPSS Statistics version 21.0.0.1 (SPSS, Inc., 2010, Chicago, IL). All values were examined to confirm that they met assumptions of normality with tests of skewness and kurtosis (<+2). Demographic variables were compared between groups (Chi-Square or two-tailed independent samples t-test) for descriptive purposes. Treatment variables and intelligence indices (VIQ and PIQ) were compared between groups with independent t-tests. A p-value of less than .05 was considered a statistically significant relationship.

Finally, we used Pearson correlation of FA values from the ROIs empirically-identified from the TBSS analyses with the standardized Verbal and Performance IQ scores to investigate the effect of lower white matter integrity of the survivors on intellectual abilities. No adjustment was made for multiple correlation analyses; instead we limited the focus to VIQ and PIQ indices. Similarly, NPS was correlated with FA values to examine the association of IQ with cumulative neurological risk factors. A p-value of less than .05 was considered a statistically significant relationship.

## Results

### Demographic and Treatment Variables of Participants

**Table 1** shows the demographic information of each participant group (RT, NRT and HC groups). No statically significant difference was observed across demographic characteristics of age, sex and education. In the RT group, significantly more survivors received chemotherapy relative to the NRT group (79% vs 8%, respectively; *χ* ^*2*^(1) = 13.72, *p* < .001). Similarly, the RT group had significantly greater proportion of patients who were treated for hormone deficiency (93% vs 15%, respectively; *χ* ^*2*^(1) = 16.39, *p* = .000). However, both groups had a non-significant difference in proportion of patients with hydrocephalus (79% vs. 62%). Using the NPS [10, 16], we quantified the cumulative neurological complications for each participant. As expected, we found that the RT group had significantly greater degree of neurological complexity (*t*(24) = -8.51, *p* = .000).

Furthermore, the RT group was significantly different from NRT and HC groups on all indices of intellectual abilities (p < .05) measured in this study, with the exception of no statistical significance between RT and NRT groups on the Similarities subtest. NRT survivors and controls performed similarly across all IQ indices and subtests with comparable standard deviations, suggesting that NRT and HC groups have similar cognitive abilities as well as a more restricted or smaller range relative to the RT group. In contrast, standard deviations are consistently higher in the RT group (e.g. FSIQ: SD = 14.63 in the RT group, compared to SD = 7.47 in the NRT group, and SD = 7.50 in the HC group), reflecting a greater variability of intellectual performance in the RT group. Interestingly, on average, all three groups performed in the average range (90–109) on IQ indices; however, the RT group was on average, in the lower part of that range.

### Differences in White Matter Integrity

#### Survivors versus Healthy Controls

**Fig 1:**
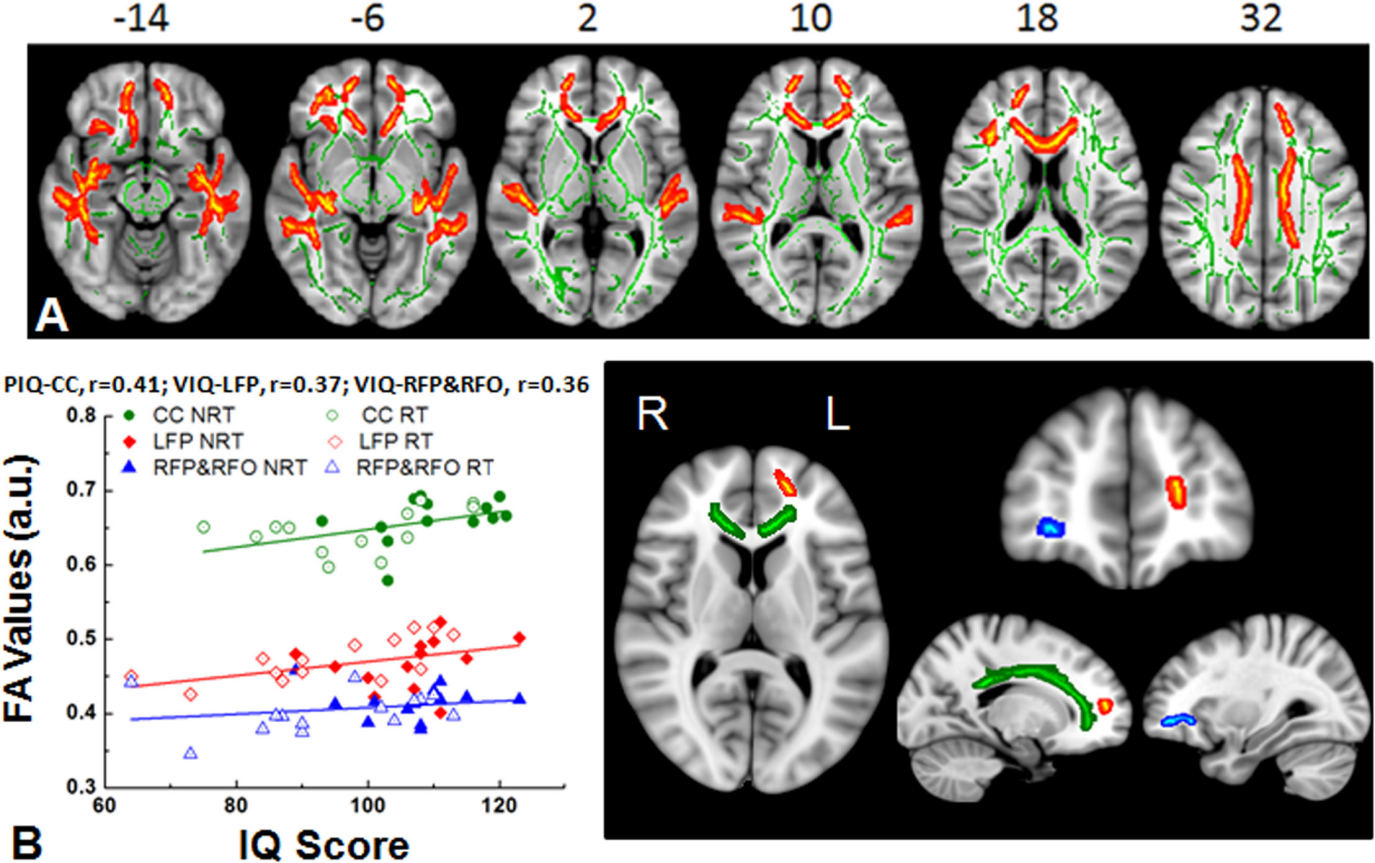
White matter differences between survivors and healthy controls. (A) Significant white matter differences between survivors (in both RT and NRT groups) and healthy controls (HC) were found in the empirically-identified white matter regions from TBSS. White matter skeleton (color coded in green) is overlaid on a T_1_ weighted image. Clusters of significantly lower fractional anisotropy (FA) for survivor group are in orange and red. (B) The plot of statistically significant correlations between intellectual performance and the white matter FA measured from the areas of left frontal pole (red), right frontal pole (blue) and corpus callosum (green). The open symbols represent survivors with radiation treatment and solid symbol represent those with no radiation treatment. LFP = left frontal pole (red), RFP = right frontal pole (blue), CC = corpus callosum (green). VIQ = verbal intelligence quotient, PIQ = performance intelligence quotient. a.u. = arbitrary units.

The white matter integrity of survivors and controls were compared based on the FA maps of whole brain white matter tract skeletons obtained from TBSS with TFCE. Lower FA was observed in several brain regions of survivors (**Fig 1**) relative to controls. More specifically, the lower mean FA in survivors was found in the corpus callosum, bilateral frontal medial, frontal pole, and middle temporal regions, also the left superior frontal, right inferior frontal and right frontal orbital regions as shown in **Fig 1A**. The obtained FA values, location and volume of brain structures with lower FA are summarized in **Table 2**.

**Table 2:** Fractional anisotropy (FA) values of white matter regions in all brain tumor survivors were lower compared to those of healthy controls, and correlated with IQ.

Structural Atlas	Volume (mm^3^)	COG [X Y Z] (mm)	Controls (FA)	Survivors (FA)	VIQ^a^	PIQ^a^
**CC**	5763	[0 13 21]	0.70 ± 0.04	0.65 ± 0.03**	0.29*	0.41**
**LSF, LFP**	684	[–12 23 50]	0.42 ± 0.03	0.38 ± 0.02**	0.21	0.28*
**LMF**	355	[–14 45 –13]	0.42 ± 0.03	0.38 ± 0.02**	0.26	0.21
**LFP**	110	[–16 53 10]	0.51 ± 0.03	0.47 ± 0.03**	0.37**	0.30*
**RMF, RFP**	809	[16 56 2]	0.53 ± 0.04	0.30 ± 0.02**	0.30*	0.31*
**RFP, RFO**	213	[30 40 –2]	0.45 ± 0.04	0.41 ± 0.03**	0.36**	0.30*
**RIF, RMF**	116	[37 25 18]	0.54 ± 0.03	0.50 ± 0.05**	0.04	0.24
**RFO**	116	[29 27 –10]	0.38 ± 0.05	0.35 ± 0.04**	0.21	0.24
**LST, LMT, LIT, LPT**	3803	[–44 –17 –13]	0.47 ± 0.04	0.42 ± 0.02**	0.34*	0.36**
**RST, RMT, RIT, RPT**	3778	[43 –19 –12]	0.48 ± 0.04	0.44 ± 0.02**	0.33*	0.32*

Note: ^a^: Correlation coefficient; FA = Fractional Anisotropy, VIQ = Verbal Intelligence Quotient; PIQ = Performance Intelligence Quotient.

*: P < 0.05

**: P < 0.01.CC: corpus callosum, LSF: left superior frontal; LFP: left frontal pole; LMF: left middle frontal; LFP: left frontal pole; RMF: right middle frontal; RFP: right frontal pole; RFO: right frontal orbital; RIF: right inferior frontal; LST: left superior; LMT: left middle temporal; LIT: left inferior temporal; LPT: left planum temporale; RST: right superior temporal; RMT: right middle temporal; RIT: right inferior temporal; RPT: right planum temporale.

To investigate the effect of lower white matter integrity on intellectual abilities, we correlated FA values from the empirically identified ROIs with the standardized Verbal and Performance IQ scores. The majority of FA values obtained from the white matter in both frontal and corpus callosum regions correlated positively with cognitive performance, such that a lower score was associated with the lower white matter integrity (**Table 2**). **Fig 1B** demonstrates the relationship of FA values with VIQ and PIQ in a couple of selected ROIs. For example, VIQ is correlated with the FA values of the white matter in left frontal pole and PIQ is positively correlated with the FA values of corpus callosum from all survivors with and without radiation treatment.

#### Survivors with Radiation Treatment with and without Chemotherapy versus Healthy Controls

**Fig 2:**
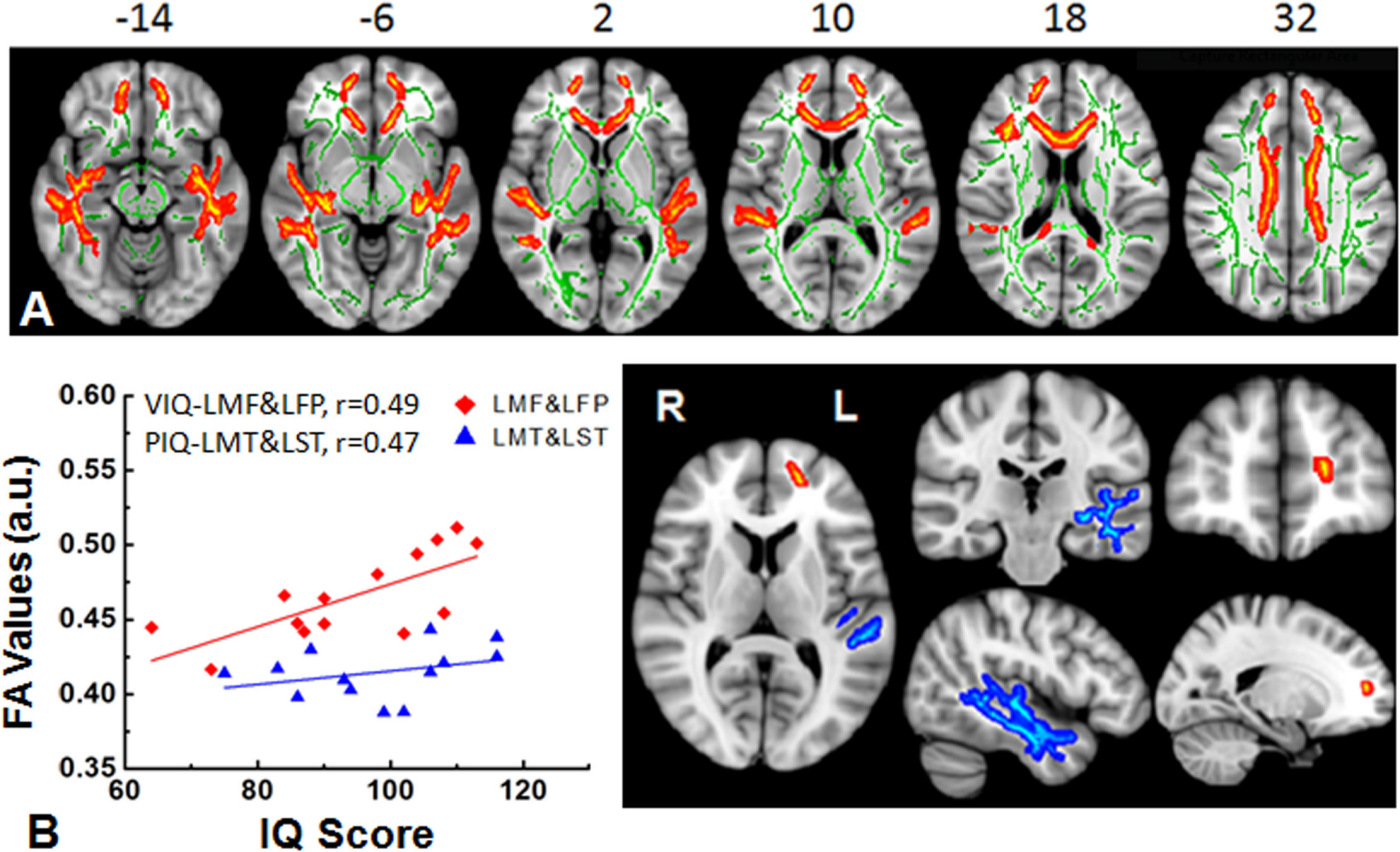
White matter differences between survivors treated with radiation therapy with or without chemotherapy and healthy controls. (A) Significant white matter differences between survivors with radiation treatment with or without chemotherapy (RT) and healthy controls (HC) were found in the empirically-identified white matter regions from TBSS. White matter skeleton (color coded in green) is overlaid on a T_1_ weighted image. Clusters of significantly lower fractional anisotropy (FA) for survivor group are in orange and red. (B) The plot of statistically significant correlations between intellectual performance and the white matter FA measured from the areas of left middle frontal (red) and left middle temporal (blue). LMF = left middle frontal (red), LMT = left middle temporal (blue). VIQ = verbal intelligence quotient, PIQ = performance intelligence quotient. a.u. = arbitrary units.

Most previous studies have examined the difference of white matter between pediatric tumor patients treated with radiation with or without chemotherapy versus clinic-referred controls (e.g., evaluated for headache without identified MRI abnormality)[5, 8, 14, 24]. We extended these earlier findings from childhood brain tumor survivors in their early years to survivors with a mean survival time of 13 years, thus focusing on long term outcomes of young adults. **Table 3**summarizes the white matter regions that showed lower FA values in survivors with the RT group in comparison to FA values of the HC group. The regions found to have lower FAs in the RT group are shown in **Fig 2A**. In addition, robust correlations were identified between FA values and VIQ or PIQ in those TBSS empirically-identified regions. The examples of correlations between IQ performance and white matter FA regions are shown in two selected regions **(Fig 2B),** where positive correlations between FA values of the left middle frontal white matter and VIQ, and FA values of left middle temporal white matter and PIQ in survivors in the RT group are presented.

**Table 3:** Fractional anisotropy (FA) values were lower in specific white matter regions in survivors compared to healthy controls, and correlated with IQ.

Groups	Structural Atlas	Volume (mm^3^)	COG [XYZ] (mm)	Control (FA)	Survivor (FA)	VIQ^a^	PIQ^a^
	**CC**	6671	[0 13 21]	0.71 + 0.04	0.66 + 0.03**	0.40**	0.47**
	**LSF**	685	[–11 30 46]	0.42 + 0.03	0.38 + 0.03**	0.29	0.39*
	**LMF**	282	[–13 52 –8]	0.46 + 0.03	0.42. + 0.03**	0.27	0.36*
**RT**	**LMF, LFP**	105	[–16 53 10]	0.50 ± 0.03	0.47 ± 0.03**	0.49**	0.44**
**vs**	**RSF**	513	[13 45 29]	0.46 ± 0.03	0.42 ± 0.02**	0.31*	0.33*
**HC**	**RMF**	601	[15 52 3]	0.44 ± 0.03	0.39 ± 0.03**	0.37*	0.42*
	**RIF**	164	[36 26 18]	0.52 ± 0.03	0.48 ± 0.05*	0.18	0.36*
	**LMT, LST**	4638	[–44 –18 –12]	0.47 ± 0.04	0.41 ± 0.02**	0.41**	0.47**
	**RMT, RST**	3443	[45 –24 –7]	0.48 ± 0.04	0.42 ± 0.02**	0.43**	0.40*
**NRT**	**CC**	4558	[0 4 20]	0.75 ± 0.03	0.71 ± 0.03**	-0.005	0.226
**vs**	**LFO, LFP**	128	[–27 31 –7]	0.50 ± 0.04	0.44 ± 0.03**	0.052	0.032
**HC**	**LIF**	92	[–35 37 4]	0.48 ± 0.03	0.44 ± 0.04**	0.009	-0.064

Note: a: Correlation coefficient; VIQ = Verbal Intelligence Quotient; PIQ = Performance IQ; FA = fractional anisotropy; RT = survivors treated with radiation treatment with or without chemotherapy; NRT = survivors who did not receive radiation treatment; HC = Healthy Controls.

*: P < 0.05

**: P < 0.01. CC: corpus callosum, LSF: left superior frontal; LMF: left middle frontal; LFP: left frontal pole; RSF: right superior frontal; RMF: right middle frontal; RIF: right inferior frontal; LFO: left frontal orbital; LFP: left frontal pole; LIF: left inferior frontal; LST: left superior temporal; LMT: left middle temporal; LIT: left inferior temporal; LPT: left planum temporale; RST: right superior temporal; RMT: right middle temporal; RIT: right inferior temporal; RPT: right planum temporale.

#### Survivors without Radiation Treatment versus Healthy Controls

**Fig 3:**
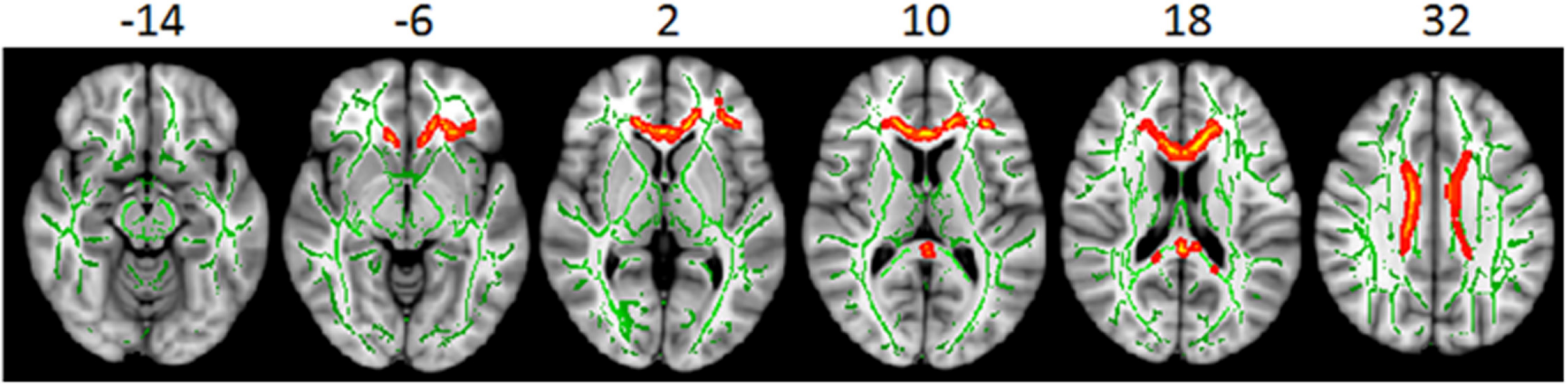
White matter differences between survivors with no radiation treatment and healthy controls. Significant white matter differences between survivors with no radiation treatment (NRT) and healthy controls (HC) were found in the empirically-identified white matter regions from TBSS. White matter skeleton (color coded in green) is overlaid on a T_1_ weighted image. Clusters of significantly lower fractional anisotropy (FA) for survivor group are in orange and red. No statistically significant correlation between intellectual performance and the white matter FA was found in these areas.

Distinct from prior studies, we compared survivors without radiation treatment to healthy controls to investigate whether the neurological complications which are common among most brain tumor survivors (e.g., brain tumor itself and hydrocephalus) may disrupt white matter development without the factor of radiation treatment. In this case, lower FA was observed in the corpus callosum and the left frontal regions (**Fig 3**) in the NRT group. Although lower FA in these two regions also was observed in the RT groups, more brain regions appear to be affected in the RT group. Taking together, these observations suggest that the white matter in corpus callosum and the left frontal area may be particularly vulnerable to damage from common neurological factors (e.g. brain tumor itself, hydrocephalus). However, no statistically significant correlations with IQ were found between these empirically identified regions (**Table 3**) in NRT and HC groups. This lack of correlation is likely due to the similar and restricted range of intellectual performance within the high average range in both NRT and HC groups (and notably smaller standard deviations compared to the RT group). Yet, it appears that FA is sensitive to white matter differences in NRT versus HC groups.

#### Survivors with Radiation Treatment with or without chemotherapy versus Survivors without Radiation Treatment

**Fig 4:**
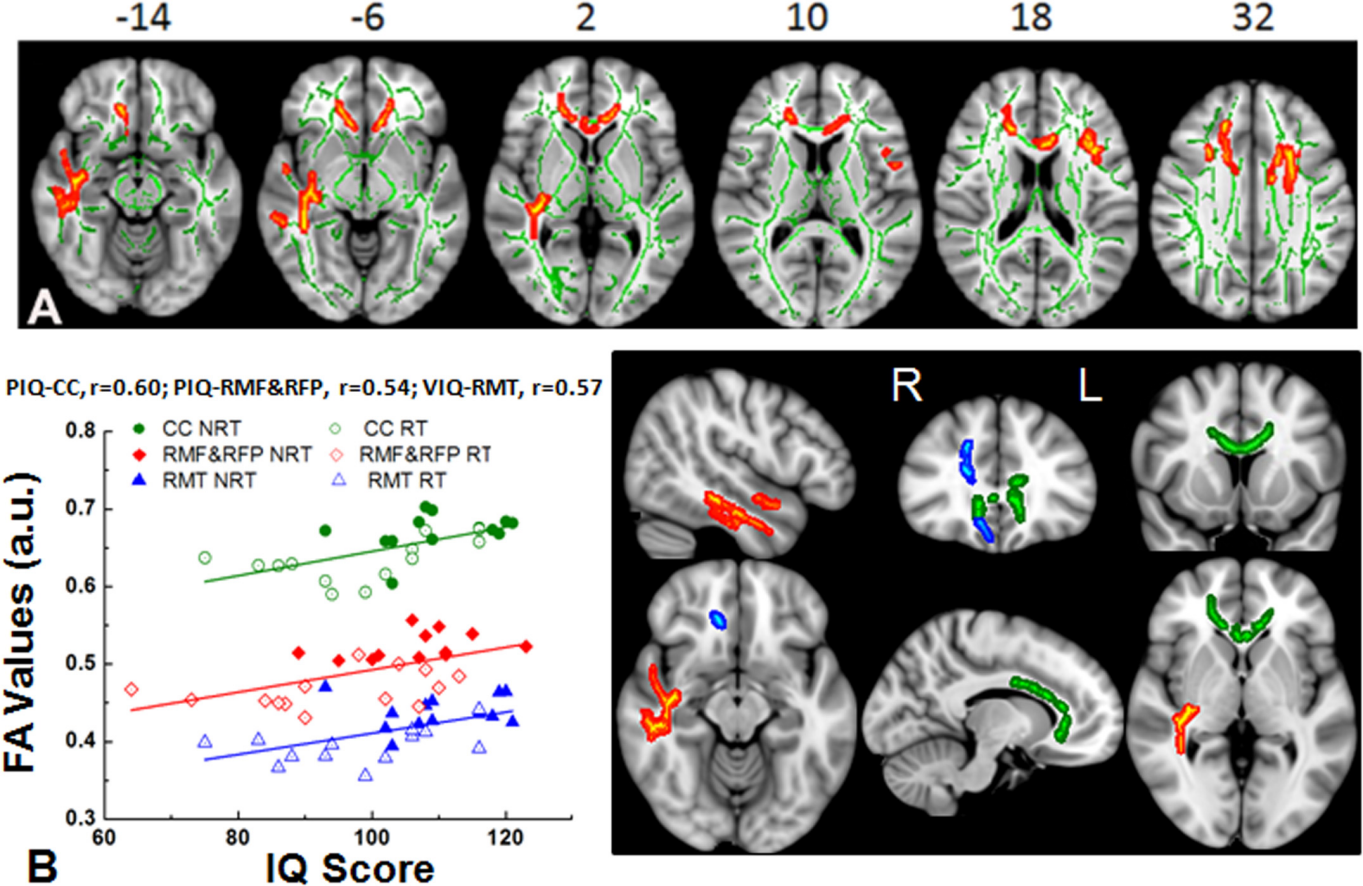
White matter differences between survivors with radiation treatment with or without chemotherapy (RT) and survivors without radiation treatment (NRT). (A) Significant white matter differences were identified between the RT group and the NRT group in the empirically-identified white matter regions from TBSS. White matter skeleton (color coded in green) is overlaid on a T_1_ weighted image. Clusters of significantly lower fractional anisotropy (FA) for the RT survivor group are in orange and red. (B) The plot of statistically significant correlations between intellectual performance and the white matter FA measured from the areas of anterior portion of corpus callosum (green), right middle temporal (red) and right middle frontal (blue) regions. CC = corpus callosum (green), RMF = right middle frontal (blue), RMT = right middle temporal (red). VIQ = verbal intelligence quotient, PIQ = performance intelligence quotient, a.u = arbitrary units.

In the cohort of the current study, the RT group presented with greater cumulative neurological complexity relative to the NRT group. This difference in cumulative neurological complexity is likely resulted from the combination of radiation treatment and chemotherapy as well as possible neuroendocrine dysfunction in addition to the common conditions shared with those without radiation treatment (e.g., surgery and hydrocephalus). To examine the effect of more complex neurological factors that coexist with radiation treatment on the quality of white matter integrity development, we analyzed FA of survivors treated with radiation and compared to survivors without radiation treatment. We found that the survivors in the RT group exhibited lower FA in the white matter of the anterior portion of corpus callosum, right middle temporal and frontal regions (**Fig 4A)** compared to the NRT group. The results of FA values are summarized in **Table 4**. In addition, very robust positive correlations were identified between IQ and FA (**Fig 4B**). Furthermore, FA values of these identified regions are positively associated with the NPS, a measure of cumulative neurological risk factors (p < .01).

**Table 4:** The correlations of IQ and cumulative neurological risk with fractional anisotropy values of specific white matter regions in survivors with radiation treatment with and without chemotherapy (RT) compared to the survivors who did not receive radiation treatment (NRT).

Structural Atlas	Volume (mm^3^)	COG [X Y Z] (mm)	NRT (FA)	RT(FA)	VIQ^a^	PIQ^a^	NPS^a^
**CC**	1969	[1 22 16]	0.67 ± 0.02	0.63** ± 0.03	0.43*	0.60**	-.657**
**RMF, RFP**	821	[20 38 26]	0.44 ± 0.02	0.39** ± 0.03	0.45*	0.54**	-.641**
**RMF**	127	[30 20 29]	0.46 ± 0.04	0.39** ± 0.04	0.50**	0.45*	-.443*
**LIF**	247	[–38 16 16]	0.50 ± 0.02	0.46** ± 0.02	0.32	0.42*	-.767**
**LMF**	318	[–25 8 32]	0.47 ± 0.03	0.43** ± 0.03	0.32	0.35	-.616**
**RMT**	1798	[44 –21 –11]	0.52 ± 0.02	0.47** ± 0.02	0.57**	0.45*	-.742**
**RTP**	133	[42 7 –24]	0.38 ± 0.04	0.33** ± 0.03	0.25	0.29	-.561**

Note: ^a^: Correlation coefficient; VIQ = Verbal Intelligence Quotient; PIQ = Performance IQ; NPS = Neurological Predictor Scale; FA = fractional anisotropy; RT = survivors treated with radiation treatment with or without chemotherapy; NRT = survivors who did not receive radiation treatment.

*: P < 0.05

**: P < 0.01. CC: corpus callosum; RMF: right middle frontal; RFP: right frontal pole; LIF: left inferior frontal; LMF: left middle frontal; RMT: right middle temporal; RTP: right temporal pole.

#### Secondary posterior fossa analyses

The majority of previous studies have focused exclusively on posterior fossa tumors. Therefore, we repeated the analyses of IQ and FA group differences, and FA and IQ correlational analyses with survivors of posterior fossa tumors only [5, 6, 8]. FA differences between the RT and NRT groups, RT and HC groups, and NRT and HC groups remained significantly different. Similarly, when comparing IQ between the three groups the same pattern of results remained. The IQ of the RT group is significantly lower than those of NRT and HC groups (VIQ:*t*(19) = 2.84, *p* = .01; *t*(36) = 3.71, *p* = .001; PIQ:*t*(19) = 2.10, *p* = .05; *t*(36) = 2.67, *p* = .01*)*. Finally, there is no appreciable change in correlations between FA and VIQ and PIQ. The similar pattern of results remained when examining the current findings in the subset of posterior fossa patients.

## Discussion

The current study provides imaging-derived evidence of white matter disruption in adult survivors of childhood brain tumors relative to demographically-matched healthy controls, which allows for determining if the disruptions of white matter integrity in these brain regions relate to intellectual performance. Our findings are generally consistent with previous DTI and volumetric studies that reported disrupted white matter and related cognitive correlates [5–9, 25, 26]. Furthermore, significant correlations of verbal and performance IQ with these TBSS-derived white matter regions **(Table 4)** illustrated the robust nature of these findings. The results from this unique cohort of long-term adult survivors of childhood brain tumors expand the findings from previous studies with short-term childhood survivors. In particular, the results from the current study revealed that the RT group presented with multiple regions with white matter disruption and that these regions were correlated with intellectual performance (**Fig 2**). The process of myelination within the white matter continues until approximately 20 years of age [27], and the presence of a neurological condition such as brain tumor during childhood appears to disrupt or possibly delay the white matter maturation. Earlier studies have suggested that possible mechanisms of radiation-induced white matter damage are gradual loss of oligodendrocytes or their precursors as well as blood-brain barrier disruption and damage to the cerebral vasculature [28, 29].

The other new findings of the current study were microstructural white matter differences between the survivor groups of RT and NRT, and NRT and HC groups. The NRT group is a critical comparison group when there is a need to differentiate the influences of the tumor, treatment and other neurological sequelae such as tumor compression of white matter when experiencing intracranial pressure or possibly disrupted white matter following removal of tumor with neurosurgery. The survivors in the NRT group included in the current study demonstrated significantly higher white matter integrity than the survivors in the RT group. The current findings are consistent with the previously reported observations that radiation treatment with or without chemotherapy is especially neurotoxic to white matter integrity.

Nonetheless, most of regions of white matter integrity difference were positively and highly-related to intellectual performance in this sample. Comparing RT and NRT groups is critical to identifying the role of more complex diagnosis and subsequent brain tumor treatment relative to more limited and circumscribed interventions that have been less associated with cognitive decline. The NRT group demonstrated significantly lower white matter integrity relative to controls in the corpus callosum and three regions in the left frontal lobe. Only two previous studies have included the NRT group in comparison [8, 11]. Our findings support the report of the significant frontal white matter differences by Rueckriegel et al. [8]; although Law and colleagues (2011) did not detect differences between NRT and controls which may be possibly due to small sample size of the NRT group. The only other known study of long-term survivors of childhood brain tumors did not have a comparison group (neither the NRT group nor controls) and also found only one correlation between FA and cognition, among 6 ROIs and multiple cognitive and self-report measures [19]. Future studies should continue to utilize a carefully-matched NRT group as well as neurologically-healthy controls.

In addition, our findings highlight that the RT group has inherently more cumulative neurological risk factors because this group also had been more frequently treated with chemotherapy and neuroendocrine interventions relative to the NRT group. These cumulative factors are not surprising as they commonly co-occur in patients with medulloblastoma and the combination of treatments may likely contribute to the improved survival. However, rarely are these other neurological factors evaluated and reported when examining the effects of radiation on white matter integrity or cognitive outcomes. Therefore, it is critical to consider these additional treatments as contributing and possibly interacting with the radiation treatment the RT group receives. In contrast, hydrocephalus, extent of surgery, and seizure medication were not differently represented between groups in the current study. It should be noted that the current study, like previous studies, is unable to claim the findings are solely due to radiation. It is possible that the pre and post-treatment disease complexity also played a role in these outcomes. It was for this reason that the cumulative nature of the neurological sequelae was examined. The cumulative NPS scores were significantly associated with less white matter integrity in the TBSS-identified regions of lower FA in survivors. The robust correlations of white matter FA with NPS further documented the cumulative effects of neurological factors that are associated with white matter disruption. Interestingly, our previous research has found that the NPS was correlated with hippocampal and putamen volumes but not whole brain volume [30] suggesting the cumulative neurological risk factors have a unique effect on subcortical volumes in adult survivors of childhood brain tumors. It is possible that brains experiencing cumulative neurological complications relative to the healthy brain contribute to a multifactorial disruption of white matter and cognition. Future prospective longitudinal studies with larger samples need to consider exploring the cumulative and possibly interactive impact of the neurological factors on white matter and cognitive development. Larger multi-site studies will be better equipped to examine the interaction between these treatments, and ideally with sophisticated measures such as dosimetry or integral biologically effective dose [31] and specific chemotherapy protocols. Likewise, it is these future and larger more sophisticated studies that will be able to tease apart the influence of different treatment factors that contribute to outcome.

The current findings must be viewed in the context of the possible limitations. Previous studies have examined white matter integrity almost exclusively in childhood medulloblastoma. However, our survivors represent a heterogeneous brain tumor group. This potential confound was addressed with secondary analyses on the FA and IQ of only those survivors with posterior fossa tumors and the results did not appreciably change. It is also possible that our current survivor groups (RT and NRT) are higher functioning individuals among long-term survivors because the current sample needed to be able to be in compliance with MRI (MRI safe and artifact free). Individuals with more complex neurological and impaired intellectual outcomes may not be able to meet the inclusion criteria for MRI. Therefore, it is important to consider this limitation across all DTI studies of brain tumor survivors, as it suggests that studies using MRI may represent the group with better outcomes with regard to white matter and/or cognitive disruptions.

In summary, this study provides quantitative imaging measurement of white matter disruption and correlation with intellectual performance in adult survivors of childhood brain tumor with radiation treatment with or without chemotherapy and those without radiation treatment. Although white matter damage in survivors without radiation treatment was less widespread relative to that measured in survivors with radiation treatment with or without chemotherapy, significant regions of white matter differences were detected in survivors without radiation treatment relative to controls. However, the cause of this disruption as to whether the lower FA is due to loss of white matter, disruption or delay of maturing white matter, or individual vulnerability to neurotoxicity associated with treatments or tumor complications needs to be further investigated in future longitudinal studies. Furthermore, the findings of this work add to our understanding of the mechanisms underlying cognitive outcomes reported in the increasing number of adult survivors of childhood brain tumor. In addition, non-invasive diffusion tensor imaging and quantitative measurement of white matter integrity could be employed as a biomarker to monitor treatment and to guide the design of novel neuroprotective agents for pediatric brain tumor patients.

## Supporting Information

S1 Table Demographic, treatment history, and intellectual performance of each group. RT = survivors who received radiation treatment with or without chemotherapy, NRT = survivors who did not receive radiation treatment. Groups were similar across demographic variables. *: Variables with significant group difference (*p* < .05). ^a,b^: Different superscripts (e.g., ^a^ and ^b^) signify significant mean differences between groups (χ2, *p* < .05), whereas matching superscripts illustrate similar means (e.g., ^b^ and ^b^). RT group had significantly more individuals treated with chemotherapy and individuals identified with hormone deficiency. Across most cognitive tasks and indices, the RT group was significantly lower relative to both NRT and HC groups; in contrast, the NRT group was similar to controls. IQ Mean = 100, SD = 15; Subtest T Score Mean = 50, SD = 10.
(XLSX)

S2 Table Fractional anisotropy (FA) values of white matter regions in all brain tumor survivors were lower compared to those of healthy controls, and correlated with IQ. Correlation coefficient; FA = Fractional Anisotropy, VIQ = Verbal Intelligence Quotient; PIQ = Performance Intelligence Quotient. *: P < 0.05, **: P < 0.01. CC: corpus callosum, LSF: left superior frontal; LFP: left frontal pole; LMF: left middle frontal; LFP: left frontal pole; RMF: right middle frontal; RFP: right frontal pole; RFO: right frontal orbital; RIF: right inferior frontal; LST: left superior; LMT: left middle temporal; LIT: left inferior temporal; LPT: left planum temporale; RST: right superior temporal; RMT: right middle temporal; RIT: right inferior temporal; RPT: right planum temporale.
(XLS)

S3 Table Fractional anisotropy (FA) values were lower in specific white matter regions in survivors compared to healthy controls, and correlated with IQ. Correlation coefficient; VIQ = Verbal Intelligence Quotient; PIQ = Performance IQ; FA = fractional anisotropy; RT = survivors treated with radiation treatment with or without chemotherapy; NRT = survivors who did not receive radiation treatment; HC = Healthy Controls. *: P < 0.05, **: P < 0.01. CC: corpus callosum, LSF: left superior frontal; LMF: left middle frontal; LFP: left frontal pole; RSF: right superior frontal; RMF: right middle frontal; RIF: right inferior frontal; LFO: left frontal orbital; LFP: left frontal pole; LIF: left inferior frontal; LST: left superior temporal; LMT: left middle temporal; LIT: left inferior temporal; LPT: left planum temporale; RST: right superior temporal; RMT: right middle temporal; RIT: right inferior temporal; RPT: right planum temporale.
(XLSX)

S4 Table The correlations of IQ and cumulative neurological risk with fractional anisotropy values of specific white matter regions in survivors with radiation treatment with and without chemotherapy (RT) compared to the survivors who did not receive radiation treatment (NRT). Correlation coefficient; VIQ = Verbal Intelligence Quotient; PIQ = Performance IQ; NPS = Neurological Predictor Scale; FA = fractional anisotropy; RT = survivors treated with radiation treatment with or without chemotherapy; NRT = survivors who did not receive radiation treatment. *: P < 0.05, **: P < 0.01. CC: corpus callosum; RMF: right middle frontal; RFP: right frontal pole; LIF: left inferior frontal; LMF: left middle frontal; RMT: right middle temporal; RTP: right temporal pole.
(XLSX)
